# The Induction of Colitis and Ileitis in Mice Is Associated with Marked Increases in Intestinal Concentrations of Stimulants of TLRs 2, 4, and 5

**DOI:** 10.1371/journal.pone.0009125

**Published:** 2010-02-09

**Authors:** Clett Erridge, Sylvia H. Duncan, Stefan Bereswill, Markus M. Heimesaat

**Affiliations:** 1 Department of Cardiovascular Sciences, University of Leicester, Leicester, United Kingdom; 2 Microbial Ecology Group, Rowett Institute of Nutrition and Health, Aberdeen, United Kingdom; 3 Institut für Mikrobiologie und Hygiene, Charité-Universitätsmedizin, Berlin, Germany; Columbia University, United States of America

## Abstract

**Background:**

Inflammatory bowel diseases (IBDs) appear to be modulated by the interaction of pathogen-associated molecular patterns (PAMPs) derived from intestinal bacteria with their respective innate immune receptors, including Toll-like receptors (TLRs). We aimed to establish if intestinal concentrations of proinflammatory bacterial ligands of TLR2, TLR4, or TLR5 may be altered in murine IBD models, and to characterize which of the major bacterial groups may contribute to each signal.

**Methodology/Principal Findings:**

PAMPs specific for TLR2 (lipopeptide equivalents), TLR4 (lipopolysaccharide equivalents), and TLR5 (flagellin equivalents) in human and murine fecal and intestinal samples were quantified using HEK-293 cells transfected with respective TLRs and calibrated with defined standard PAMPs. The induction of colitis in mice by dextran-sodium-sulphate treatment significantly increased colonic lipopeptide (fourfold) and LPS equivalent (550-fold) concentrations, while flagellin equivalent concentrations remained similar. The induction of ileitis by oral infection with *Toxoplasma gondii* dramatically increased ileal concentrations of lipopeptide (370-fold), LPS (3,300-fold), and flagellin equivalents (38-fold), all P<0.01. Analysis of representative strains of the major bacterial groups of the human intestine revealed that enterobacterial species are likely to be more significant contributors of soluble TLR2 and TLR4 stimulants to the intestinal milieu than *Bacteroides* species or Gram-positive Firmicutes.

**Conclusions/Significance:**

We conclude that the induction of colitis or ileitis in mice is associated with significant disease-specific alterations to the PAMP profile of the gut microbiota.

## Introduction

Inflammatory bowel diseases (IBDs), including ulcerative colitis and Crohn's disease, are widely believed to be driven by inappropriate responses to the intestinal microbiota [1]. Among the mechanisms by which bacteria may promote inflammatory signalling, recent evidence suggests that pathogen-associated molecular patterns (PAMPs) derived from intestinal bacteria may modulate IBDs via stimulation of their respective innate immune receptors, including Toll-like receptors (TLRs) [2]. TLRs, like other innate immune receptors, detect conserved PAMPs that are expressed not only by pathogenic bacteria, but also commensal bacteria, and thus serve as key sentinels for the detection of microbial products. For example, TLRs 2, 4 and 5 are the major cell-surface sensors of bacterial lipopeptides, lipopolysaccharides (LPS) and flagellins, respectively, while TLRs 3, 7, 8 and 9 detect nucleic acid motifs [3].

In most cell types, the detection of PAMPs by their respective TLR evokes a potent pro-inflammatory response, involving rapid induction of myeloid-differentiation factor 88 (MyD88) and NF-κB-dependent signalling pathways, and the resultant expression of a broad array of pro-inflammatory adhesion molecules, chemokines and cytokines. By contrast, PAMP-recognition by intestinal epithelial cells has been shown to lead to the promotion of barrier enhancement, epithelial repair and the secretion of anti-microbial peptides, rather than overt inflammatory reponses [4]–[6]. Mechanisms such as these therefore enable the healthy gut mucosa to remain largely uninflammed despite chronic lumenal exposure to large quantities of potentially pro-inflammatory PAMPs derived from the host commensal microbiota.

Several lines of evidence suggest that dysregulation of this tolerance to intestinal TLR-stimulants, or disruption of the epithelial barrier separating PAMPs from responsive underlying tissues, may contribute to the development or perpetuation of IBDs. For example, although it is well established that the presence of luminal PAMPs is not sufficient to initiate IBDs in animal models [7]–[9], it has been shown that TLR-mediated detection of some lumenal PAMPs can exacerbate existing disease. Administration of LPS or CpG-ODN (a TLR9 agonist) to rabbits or mice was shown to enhance experimentally-induced colitis or ileitis [8], [10]. Likewise, we recently reported that oral administration of the TLR4 agonist lipid-A aggravated immunopathology in a murine model of *Toxoplasma gondii*-induced ileitis [11], and that genetic deletion of TLR4, or treatment with the LPS scavenger polymyxin-B, ameliorated disease symptoms in this model [12]. In two models of spontaneous colitis, mice genetically deficient in IL-10 or intestinal epithelial cell expression of NEMO (IKK-γ), it was shown that additional deletion of the TLR-signalling adaptor protein MyD88 prevented disease progression [13], [14]. Bacterial flagellin has also been implicated in IBDs, as a functional polymorphism in TLR5 was shown to negatively correlate with Crohn's disease [15] and commensal-derived flagellin has been identified as a dominant antigen in this disease [16].

Paradoxically, however, there is also evidence that TLRs play a protective role in gut defence. For example, PAMP-sensing by intestinal epithelial cells has been reported to induce the expression of bactericidal and barrier-enhancing mediators [4]–[6], and deficiency in TLR2, TLR4 or MyD88 was shown to result in increased mortality following DSS-induced colitis [17], [18]. Deletion of TLR5 (but not other TLRs or MyD88) was also reported to lead to spontaneous colitis, but interestingly this was reversed by concurrent deletion of TLR4 in double knockout animals [19], suggesting that specific PAMPs can serve as either protective or damaging agents in different IBD models, depending on the context of their exposure.

Such findings lend useful insight to the observation that marked shifts in bacterial populations, which could alter the profile of intestinal PAMP concentrations, have been observed in human IBDs and in animal models of these diseases. In human subjects, for example, reductions in bacterial diversity are observed in active IBD [20], and Gram-negative bacteria, particularly *E. coli*, have been reported to accumulate at sites of inflammation [21]–[24]. Likewise, we showed recently using both culture-dependent and culture-independent techniques that marked enterobacterial overgrowth occurs in murine models of *T. gondii*-induced ileitis and DSS-induced colitis [11]–[13].

Taken together, these results suggest that TLR-mediated recognition of intestinal PAMPs plays a complex but key role in the modulation of IBD pathology. As very little information is currently available regarding which bacterial groups may contribute to the soluble and bacteria-associated PAMP pools in the intestine, or of how PAMP concentrations in the intestinal contents may be altered during the course of IBDs, we aimed to quantify the relative biological activities of PAMPs specific for TLR2 (lipopeptide-equivalents), TLR4 (lipopolysaccharide-equivalents) and TLR5 (flagellin-equivalents) in human and murine faecal and intestinal samples, and to establish whether the concentrations of these agents may be altered in two murine models of IBDs, *T. gondii*-induced ileitis and DSS-induced colitis.

## Materials and Methods

### Ethics Statement

All animal experiments were conducted according to German animal protection laws (LAGeso Berlin, G0170/04) with approval from the Charité-Universitätsmedizin ethical committee. Animal welfare was monitored by daily assessment of total clinical scores using combined data of weightloss, occurance of blood in stool, and stool consistence. Faecal samples from healthy human volunteers were collected with written informed consent, although as no advertisement, reward, intervention or invasive procedures were involved, institutional review was not sought for collection of these samples, according to local ethical guidelines.

### Cells and Reagents

Human embryonic kidney (HEK)-293 cells (ECACC Cat no: 85120602) and murine RAW 264.7 macrophages (ECACC Cat no: 91062702) were cultured in DMEM/10% FCS (Sigma). Flagellin of *Salmonella typhimurium* and the synthetic bacterial lipopeptide analogue Pam_3_CSK_4_ were from Invivogen. *Escherichia coli* R1 (NCTC-13114) derived LPS (a kind gift of Professor Ian Poxton, University of Edinburgh), was repurified by phenol re-extraction to remove TLR2-stimulating lipopeptide contaminants as described previously [25].

### Murine Models of Colitis and Ileitis

For induction of colitis, C57BL/10ScSn mice bred under specific pathogen-free (SPF) conditions were treated with 3.5% (wt/vol) dextran-sodium sulphate (DSS, 40,000 kDa, MP Biomedicals, Illkirch, France) in drinking water ad libitum for seven days. Mice received water without DSS for 24 hours before sacrifice with halothan. Colon contents were then surgically removed under sterile conditions as described previously [13]. For induction of ileitis, C57/BL6 mice were infected perorally with 100 *Toxoplasma gondii* cysts in 0.3 ml PBS by gavage as described previously [12]. The contents of ∼1 cm of the terminal ileum were removed under sterile conditions after 8 days and resuspended in PBS. Samples were then processed for measurement of soluble PAMP concentrations as described below.

### Preparation of Murine and Human Faecal PAMP Extracts

Murine colon or ileal contents were mixed 1∶4 (weight:volume) with sterile phosphate-buffered saline (PBS) and vortexed briefly. This mixture was then centrifuged at 13,000 g for 20 minutes to pellet bacteria and other large particles before decanting the supernatant for filtration using a 0.45 µm filter (Millipore) to yield sterile-filtered faecal extract (SFE), intended to be representative of the soluble PAMPs present in the gut lumen. Faecal samples from six healthy, human volunteers (age 22–33 years, 5 M, 1 F) were also processed in the same manner, although a separate aliquot of non-filtered, resuspended material was heat-treated at 100°C for 15 minutes to represent the intact, heat-killed normal flora (HKF), intended to reflect the insoluble PAMPs present in the intestine. Sterility of HKF and SFE was verified by streaking on LB-agar plates. Protein content of SFE samples was assessed by Bradford assay and adjusted to 2 mg/ml by addition of PBS. SFE concentration is presented as concentration of protein in SFE samples throughout.

### Measurement of Relative Biological Activities of PAMPs in HKF and SFE

Transfection of TLR-deficient HEK-293 cells was used to measure relative biological activities of PAMP-equivalents. Cells were plated in 96-well plates at 2×10^4^ cells per well and transfected after 24 h using Genejuice (Novagen). Amounts of construct (prepared as described in [26]) per well were 30 ng of human TLR2, TLR4 (co-expressing MD-2) or TLR5 (Invivogen), 30 ng of CD14, 20 ng of renilla luciferase-reporter construct and 10 ng of firefly luciferase-reporter construct driven by the NF-κB dependent E-selectin promoter (pELAM). 3 days after transfection, cells were challenged in triplicate with indicated concentrations of defined TLR-ligands, whole heat-killed bacteria, HKF, SFE or sterile-filtered bacterial supernatants for 18 h. NF-κB-dependent reporter expression was measured using Promega Dual-Glo reagent and normalised to co-transfected renilla expression. Fold induction was calculated relative to cells cultured in medium alone and a standard curve was prepared by plotting fold NF-κB induction vs concentration for each standard PAMP. For determination of relative PAMP abundance in HKF and SFE, samples were diluted until the induction of reporter occurred within the linear range of the standard curve. PAMP abundance was then calculated as ng per g faeces (wet-weight) for HKF, or per mg protein for soluble extracts. PAMP standards did not induce signalling in cells expressing heterologous TLRs, or in cells transfected with CD14 alone ([27] and data not shown).

Notably, because it is has been proposed that diverse ligands beyond lipopeptide- and LPS-based molecules may also contribute to signalling via TLRs [28], the TLR-stimulating capacity of each extract is expressed as a biological activity relative to that of a chosen standard lipopeptide, LPS or flagellin. For this reason, the concentration of TLR-stimulants in each sample is presented as “PAMP-equivalents” rather than *de facto* lipopeptides, LPSs or flagellins. For example, 2 µg per g “LPS-equivalent” means that each gramme of the extract has a biological activity equivalent to that of 2 µg *E. coli* R1 LPS in terms of its capacity to stimulate TLR4-signalling, and that this activity may derive from multiple potential TLR-ligands within the sample.

### Characterisation of Molecular Species Responsible for TLR-Stimulation in SFE

To determine whether lipopeptides, LPS or flagellin may be responsible for the stimulation of TLR2, TLR4 or TLR5 respectively, SFE was treated with lipases, polymyxin-B or proteinase-K. For lipase treatment, 10 µg/ml human SFE was treated with *Candida antarctica* lipase immobilised to inert acrylic beads (Sigma) for 18 h at 37°C. Beads were then pelleted by centrifugation and the supernatant was sterile filtered prior to assay using HEK-293-TLR2 cells. To examine LPS-signalling, 10 µg/ml human SFE, or 100 ng/ml *E. coli* LPS, was treated with 10 µg/ml polymyxin-B or 1 µg/ml lipid-IVa for 10 minutes before measurement of capacity to induce TLR4-signalling using HEK-293-TLR4 cells. To determine if the TLR5 stimulants in SFE are of protein origin, human SFE (10 µg/ml), or 500 ng/ml *S. typhimurium* flagellin was treated with proteinase-K (Sigma) for 4 h before enzyme denaturation at 80°C for 10 minutes.

### Macrophage Stimulation

RAW 264.7 macrophages were plated at 5×10^4^ cells per well of 96-well plates and challenged with indicated dilutions of SFE in triplicate. Supernatant TNF-α was measured at 4 h by L929 bioassay as described previously [29]. For measurement of IκBα degradation and p38 MAPK phosphorylation, cells were plated at 8×10^5^ cells per well in 6-well plates and treated with 20 µg/mL SFE from each volunteer for 30 minutes. Cellular lysates were then separated on 12% polyacrylamide gels, blotted to nitrocellulose membranes and probed with rabbit-anti-human-IκBα (SantaCruz, sc371), mouse-anti-phospho-p38 MAPK (Cell Signal, 9216S) or rabbit-anti-human-GAPDH (SantaCruz, sc25778).

### Preparation of Heat-Killed Bacteria and Supernatants

Strains of bacteria examined were: *Eubacterium rectale* (A1-86), *Eubacterium hallii* (L2-7), *Eubacterium cylindroides* (T2-87), *Butyrivibrio fibrisolvens* (16/4), *Ruminococcus obeum*-like (Sr1/5), *Faecalibacterium prausnitzii* (S3L/3), *Megamonas hypermegale* (Art12/1), *Bacteroides distasonis* (20701), *Bacteroides eggerthii* (20697) and *Bacteroides thetaiotaomicron* (B5482). *Bacteroides fragilis* (NCTC-9343) and *Bifidobacterium bifidum* cells and supernatant were kind gifts of Professor Ian Poxton (University of Edinburgh). University of Strathclyde teaching laboratory reference strains of *Escherichia coli* (NCTC-13114), *Salmonella typhimurium*, *Pseudomonas aeruginosa*, *Klebsiella pneumoniae*, *Proteus vulgaris*, *Staphylococcus aureus*, *Enterococcus faecalis*, *Streptococcus pyogenes* and *Lactobacillus plantarum* (NCIMB-6376) were kind gifts of David McNeill (University of Strathclyde). Each strain was grown in respective standard broths and conditions to late log-phase and centrifuged to harvest bacterial cells (13,000 g for 5 minutes). The remaining supernatant (*ie* the growth medium from each culture) was sterile filtered and stored at −20°C prior to assay. Bacterial cell pellets were then washed and re-suspended in sterile PBS to an optical density of 1.0 at 600 nm (corresponding to approximately 10^9^ bacteria per ml).

### Statistical Analysis

Differences in reporter activity or cytokine levels in treated cells were compared with untreated cells using one-way ANOVA followed by Dunnet's test. Intestinal PAMP concentrations are reported in the results as medians of absolute values. For statistical analysis of differences between intestinal PAMP concentrations, mean log_10_-transformed values were compared using the Student's T-test. Differences were assumed to be significant at *P*<0.05.

## Results

### Intestinal PAMP Profile Is Markedly Altered During *T. gondii*-Induced Ileitis

**Figure 1:**
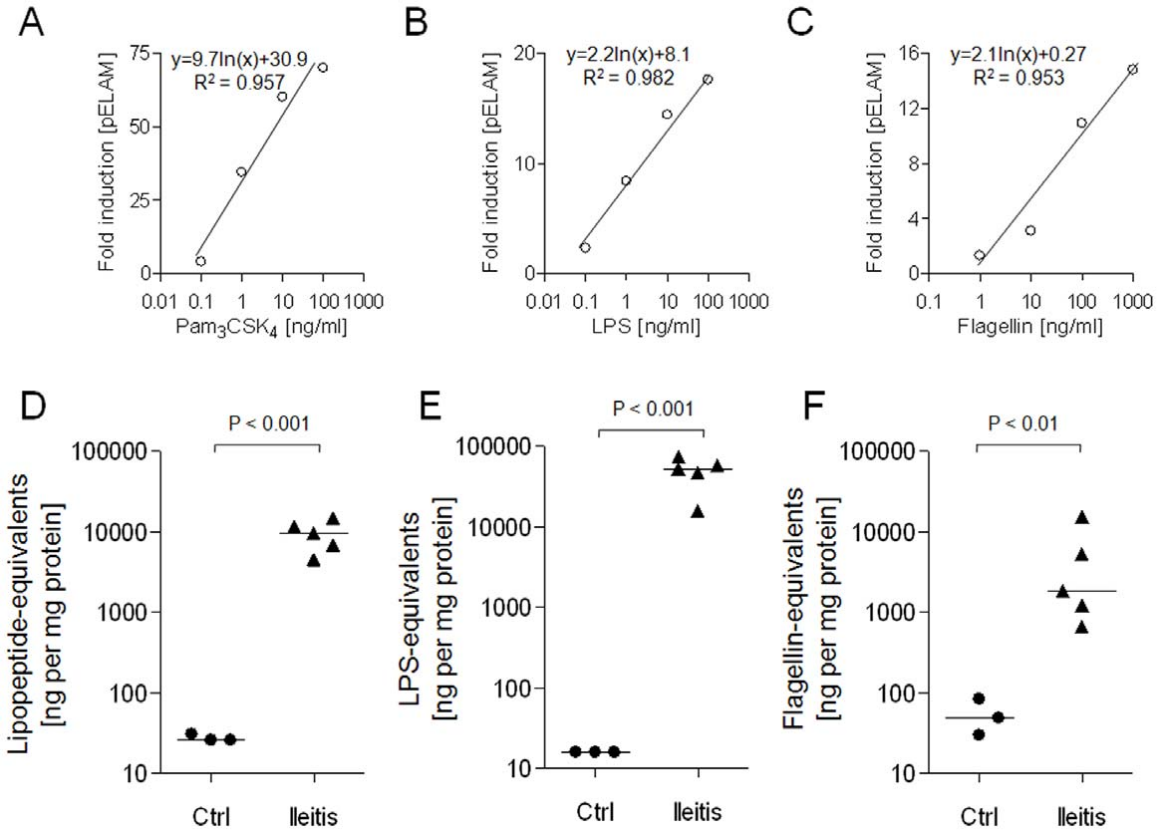
Effect of *T. gondii*-induced ileitis on PAMP profile of murine ileal contents. HEK-293 cells were transfected with CD14, NF-κB sensitive reporter construct and TLR2, TLR4/MD2 or TLR5 and challenged with indicated concentrations of Pam_3_CSK_4_, *E. coli* LPS or *S. typhimurium* flagellin, respectively, for 18 h prior to measurement of reporter activation. Typical standard curves are shown with line-fit equations and coefficients of correlation (A–C). For measurement of PAMP-equivalent concentrations in test samples, appropriate dilutions of samples were performed until fold-induction of reporter was within the range of the standard curve. SFE was then prepared from ileal contents of uninfected control mice (n = 3) or from mice in which inflammation of the small intestine was induced by oral administration of *T. gondii* (n = 5). The relative abundances of Pam_3_CSK_4_- (D), LPS- (E) and flagellin-equivalents (F) present in each SFE sample were quantified and are presented as relative concentrations of Pam_3_CSK_4_, *E. coli* LPS or *S. typhimurium* flagellin equivalents.

To determine whether intestinal PAMP concentrations may be altered in IBD, we first compared the relative abundances of TLR2, TLR4 and TLR5 stimulants in the ileal contents of healthy mice with those of mice in which inflammation of the small intestine was induced by oral administration of *T. gondii* [12]. This protocol, which results in severe Th1-type inflammation of the small intestine, has been described in detail previously [11], [12]. PAMP concentrations in ileal extracts were quantified by measuring their capacity to induce NF-κB signalling in HEK-293 cells transfected with TLR2, TLR4/MD2 or TLR5, using standard curves callibrated with Pam_3_CSK_4_, LPS and flagellin, respectively (examples of typical standard curves are shown in Figure 1A–C). This analysis revealed that all three classes of PAMP were of relatively low abundance in the ileum, presumably reflecting the low bacterial numbers in this part of the gut. However, the induction of ileitis was associated with dramatically increased concentrations of each of the PAMPs examined (Figure 1D–F). The median concentration of lipopeptide-equivalents increased from 26 to 9,694 ng/mg protein (P<0.001), LPS-equivalents from 16 to 52,688 ng/mg protein (P<0.001), and flagellin-equivalents from 49 to 1,839 ng/mg protein (P<0.01), representing fold-increases of ∼370x, ∼3,300x and ∼38x, respectively.

### Colonic PAMP Profile Is Significantly Altered During DSS-Induced Colitis

**Figure 2:**
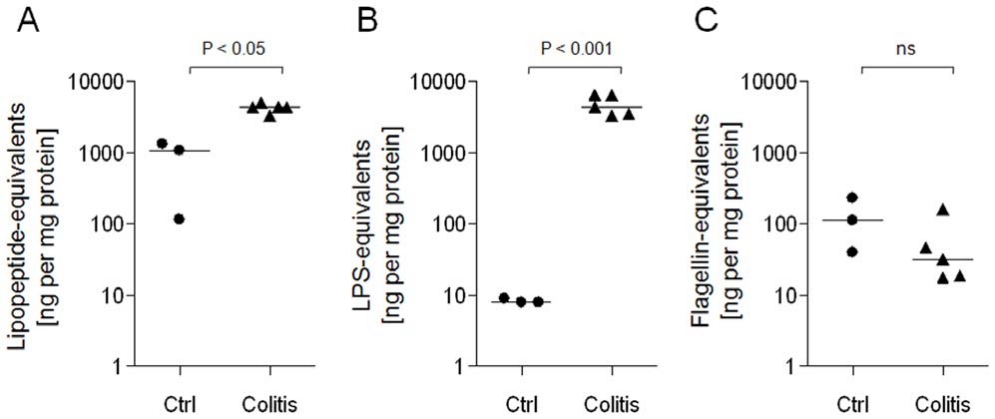
Effect of DSS-induced colitis on murine colonic PAMP profile. SFE was prepared from colon contents of untreated control mice (n = 3) or from mice in which colonic inflammation was induced by 7 days DSS-treatment (n = 5). The relative abundances of Pam_3_CSK_4_- (A), LPS- (B) and flagellin-equivalents (C) present in each SFE sample were quantified as described in the Materials and Methods.

We next sought to determine whether intestinal PAMP concentrations may also be altered in DSS-induced colitis. In healthy mice, colonic soluble LPS concentrations were similar to those measured earlier in the terminal ileum. However, lipopeptide concentration increased ∼40-fold and flagellin increased ∼25-fold in the transition from ileum to colon in healthy mice (Figure 1, 2). The induction of colitis by DSS-treatment further increased median colonic lipopeptide-equivalent concentrations from 1,083 to 4,364 ng/mg protein (P<0.05), and LPS-equivalents from 8 to 4,421 ng/mg protein (P<0.001), although colonic flagellin-equivalents remained not-significantly altered (113 vs 32 ng/mg protein, P = 0.19). These represent fold-changes of ∼4x, ∼550x and ∼0.28x, respectively, following induction of colitis (Figure 2A–C).

### Determination of Relative PAMP Abundances in Healthy Human Faecal Microbiota

**Figure 3:**
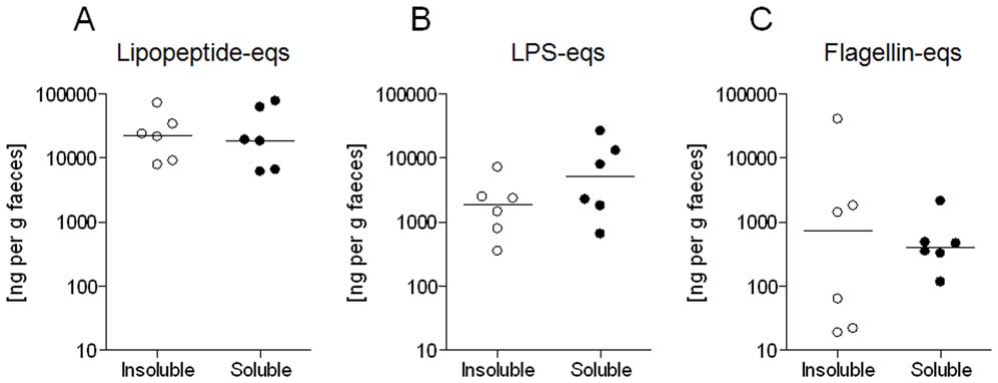
Relative abundance of PAMP-equivalents in normal human faecal flora. The relative abundances of PAMP-equivalents specific for TLR2 (A), TLR4 (B) and TLR5 (C) were measured in the heat-killed faecal flora from six healthy human subjects (insoluble fraction), or in SFE (soluble fraction) from the same subjects, using TLR-transfectants as described in the Materials and Methods.

We next measured the relative biological activities of PAMPs present in the heat-killed microbiota derived from faeces of six healthy human subjects (Figure 3A–C). The median concentration of Pam_3_CSK_4_-equivalent TLR2-stimulants was found to be approximately 10-fold higher than that of *E. coli* LPS-equivalent TLR4-stimulants (23 µg per g faeces vs 1.9 µg per g, P<0.001). Interestingly, the insoluble flagellin content of the faecal microbiota was found to be highly variable between subjects, ranging from 0.019 to 41 µg per g (Figure 3A–C).

In order to determine whether PAMPs may also exist in appreciable quantities in the soluble, *ie* non-bacterial-cell-associated, form in the human intestine, sterile filtered extracts (SFE) prepared from faeces of the same subjects were examined. Soluble PAMP concentrations were found to be similar to the concentrations measured in the insoluble fraction in each case (P = ns). For example, median concentrations of soluble lipopeptide-, LPS- and flagellin-equivalents were 19, 5.1 and 0.4 µg per g faeces, respectively (Figure 3A–C). After adjustment for protein concentration, faeces of healthy human subjects therefore contained similar concentrations of lipopeptide, but approximately 10-fold higher LPS (P<0.05) and 10-fold lower flagellin (P<0.05) than the colon contents of healthy mice (Figure 2).

### Characterisation of TLR-Stimulants Present in Human Soluble Faecal Extract

**Figure 4:**
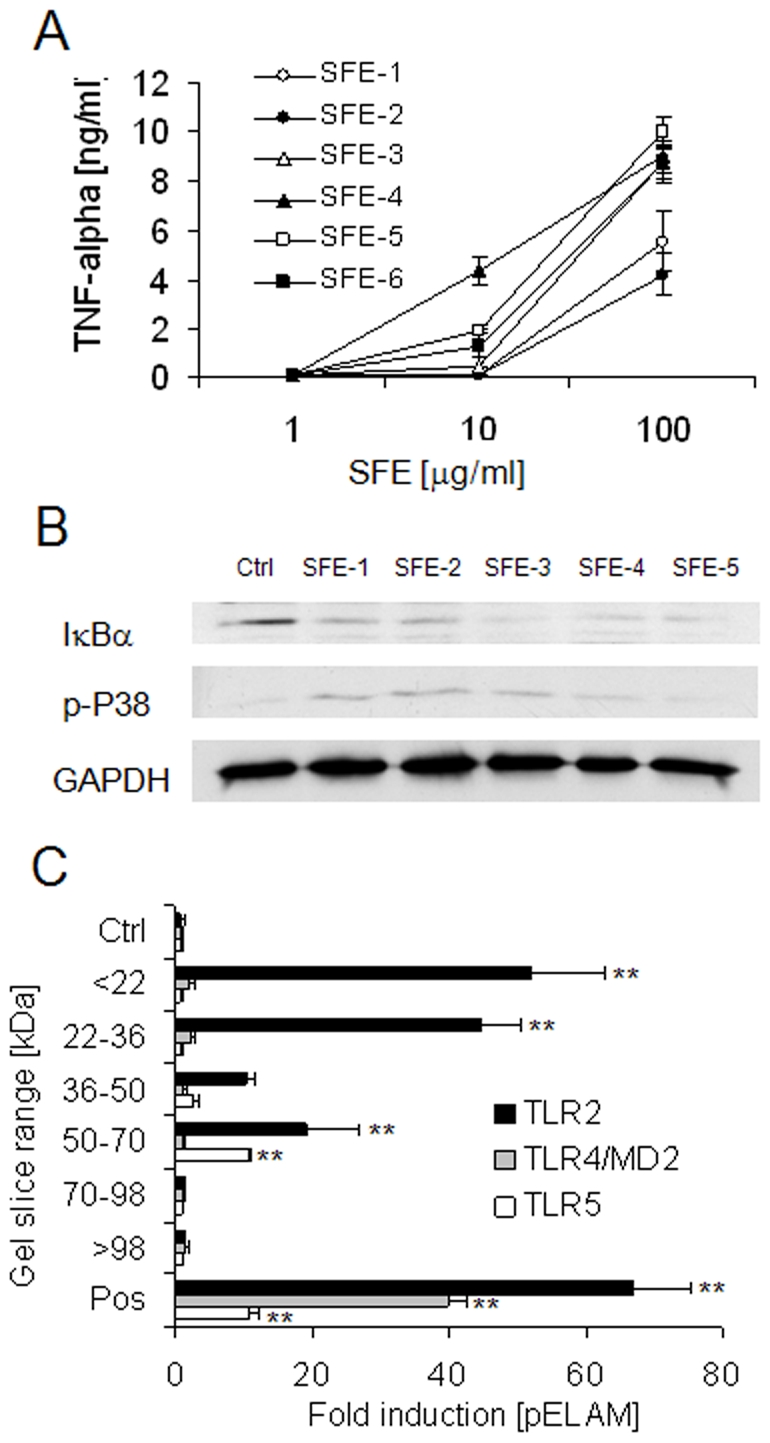
Biological activity of soluble PAMPs present in normal human faeces. (A) RAW macrophages were challenged with 1, 10 or 100 µg/ml SFE of six healthy subjects and TNF-α was measured at 4 h. Mean +/− SD shown. (B) RAW macrophages were challenged with 20 µg/ml sterile-filtered extract (SFE) of 5 healthy human volunteers. Degradation of IκBα and phosphorylation p38 MAPK were examined at 30 minutes by western blot. (C) SFE (20 µg total protein) of three healthy subjects was separated by non-reducing SDS-PAGE and PAMPs were eluted from gel-slices corresponding to indicated molecular weight ranges in PBS. HEK-293 cells transfected with TLR2, TLR4/MD2 or TLR5 were challenged with each eluate diluted 1:25 in culture medium. Responses are displayed as mean fold induction of NF-κB reporter (pELAM) +/− SD. ** P<0.01 vs cells cultured in medium alone.

The biological activity of the soluble PAMPs present in human SFE was confirmed by the observation that RAW 264.7 macrophages demonstrated TNF-α secretion, IκBα degradation and p38 MAPK phosphorylation in response to as little as 10 µg/ml SFE in a manner that was quite variable between subjects (Figure 4A–B). Separation of PAMPs in SFE by non-reducing SDS-PAGE showed that while TLR5-stimulants were of relatively high molecular weight (∼50–70 kDa), lipopeptides were typically of lower molecular weight (mainly <36 kDa), and TLR4-stimulants could not be detected in gel slice eluates (Figure 4C).

**Figure 5:**
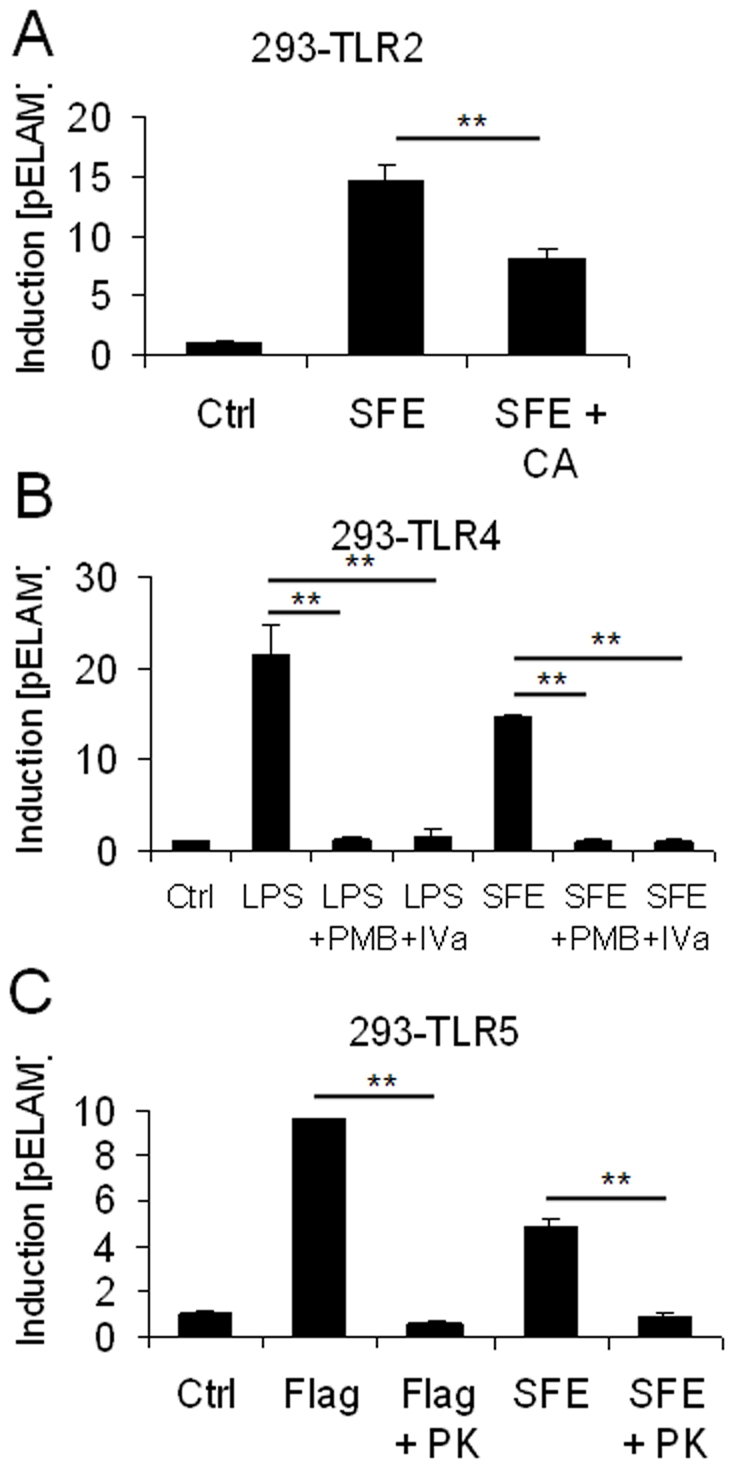
Characterisation of TLR-stimulants present in human soluble faecal extract. (A) Human SFE (10 µg/ml) was treated with immobilised lipase for 18 h at 37°C, and then assayed for potential to stimulate TLR2 signalling in HEK-293-TLR2 cells. (B) Human SFE (10 µg/ml), or 100 ng/ml *E. coli* LPS, was treated with 10 µg/ml polymyxin-B or 1 µg/ml lipid-IVa for 10 minutes before addition to HEK-293-TLR4 cells to measure capacity to induce TLR4-signalling. (C) Human SFE (10 µg/ml), or 500 ng/ml *S. typhimurium* flagellin was treated with proteinase-K for 4 h before enzyme denaturation at 80°C for 10 minutes, before application to HEK-293-TLR5 cells to measure capacity to induce TLR5-signalling.

Next, as it has been suggested that other molecules of microbial, and even host, origin could also stimulate TLR2 or TLR4 signalling in addition to lipopeptides and LPS [28], we sought to further clarify which types of molecules may be responsible for stimulating TLR2, TLR4 and TLR5 signalling in human SFE. First, as covalent acylation of lipopeptides is required for their biological activity [30], we treated human SFE with immobilised lipase. This protocol significantly reduced the TLR2-stimulating capacity of SFE, suggesting that lipopeptides are a major contributor to the intestinal pool of soluble TLR2 stimulants (Figure 5A). We then took two approaches to determine if LPS may represent the TLR4-stimulating signal observed in SFE. Treatment of SFE with polymyxin-B, which sequesters LPS from TLR4/MD2, or lipid-IVa, which competitively binds to TLR4/MD2 and thereby blocks LPS signalling, both completely blocked SFE-induced TLR4-signalling (Figure 5B), suggesting that LPS is the sole TLR4 stimulant present in SFE. Finally, we found that proteinase-K treatment blocked completely TLR5-signalling induced by flagellin or SFE, suggesting that the TLR5 stimulant in SFE is a protein, most likely flagellin (Figure 5C).

### PAMP Expression by Cultured Representatives of Major Gut Bacterial Groups

**Figure 6:**
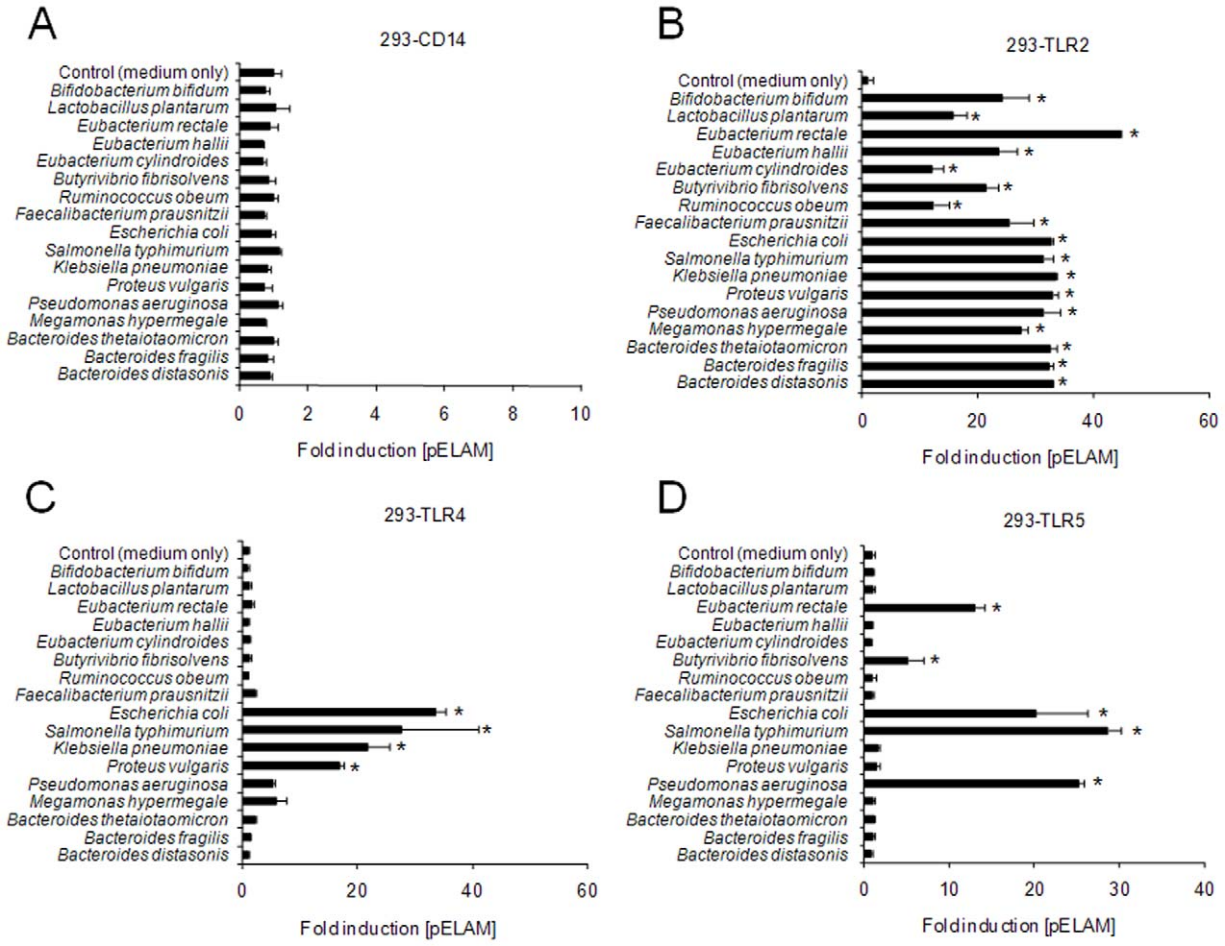
Profiles of TLR-stimulation Induced by representative gut bacterial strains. Heat-killed bacteria of indicated species were applied to HEK-293 cells transfected with CD14, NF-κB sensitive reporter construct and TLR2, TLR4/MD2 or TLR5 at a concentration of ∼10^7^ cells per ml. Responses are displayed as mean fold induction of NF-κB reporter (pELAM) +/− SD relative to cells cultured in medium alone. * P<0.05 vs medium alone.

As the expression of PAMPs specific for TLRs 2, 4 and 5 by many of the major gut-bacterial groups has not yet been established, we aimed to characterise which PAMPs may be expressed by representatives of the major gut-bacterial groups and related organisms. HEK-293 cells transfected with CD14 and reporter alone (*ie* without TLRs) were insensitive to all of the bacteria examined, indicating the specificity of each TLR-transfection assay (Figure 6A). As expected, every bacterial strain examined stimulated robust TLR2-dependent signalling, confirming the notion that the majority of bacteria express some form of lipopeptide (Figure 6B). TLR4-dependent signalling was observed in response to all of the enterobacterial species examined (*E. coli*, *S. typhimurium*, *K. pneumoniae* and *P. vulgaris*) (Figure 6C). Notably, however, no TLR4-dependent signalling was observed in response to any of the *Bacteroides* strains examined, including *B. thetaiotaomicron*, *B. fragilis* and *B. distasonis*. TLR5-dependent signalling was observed in response to the expected motile Gram-negative bacteria (*E. coli*, *S. typhimurium*, *P. aeruginosa*), but also to *Eubacterium rectale* and *Butyrivibrio fibrisolvens*, suggesting that flagella of motile Gram-positive organisms present in the gut may also stimulate TLR5 signalling (Figure 6D).

### Comparative Shedding of Soluble PAMPs by Gram-Negative and Gram-Positive Organisms

**Figure 7:**
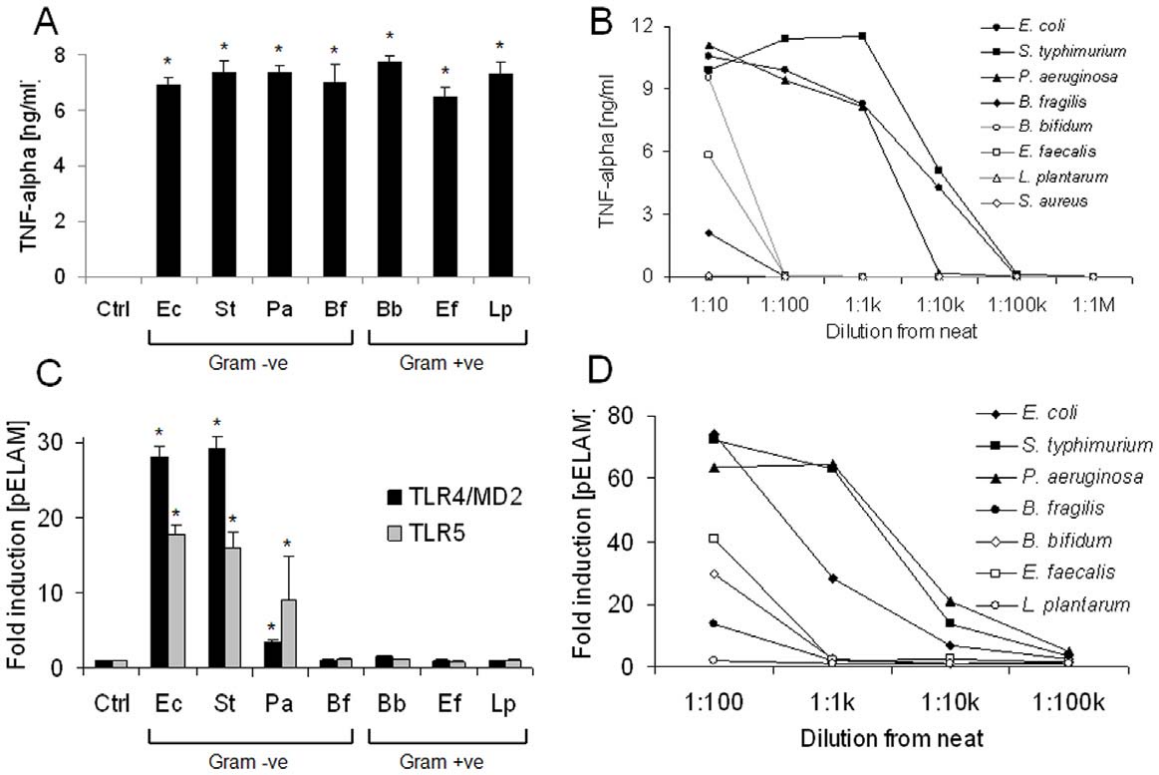
Soluble PAMP secretion by Gram-positive and Gram-negative organisms. (A) TNF-α release was measured from RAW macrophages exposed to whole heat-killed bacteria from a panel of representatives (Gram -ve: *E. coli* (Ec), *S. typhimurium* (St), *P. aeruginosa* (Pa), *B. fragilis* (Bf); Gram +ve: *B. bifidum* (Bb), *E. faecalis* (Ef), *L. plantarum* (Lp), *S. aureus* (Sa)), at a concentration of ∼10^7^ bacteria per ml. (B) Serial ten-fold dilutions of sterile-filtered supernatants from each bacterial culture were made in DMEM/10% FCS, added to RAW macrophages in triplicate and TNF-α release was measured at 4 h. (C) HEK-293 cells transfected with TLR4/MD2 or TLR5, were challenged with sterile filtered growth supernatants (1∶100). (D) HEK-293 cells transfected with TLR2 were challenged with 10-fold serial dilutions of each sterile-filtered supernatant. Data shown are representative of at least three independent experiments. * P<0.01 vs cells cultured in medium alone.

As it is possible that soluble PAMPs may be more likely to cross damaged epithelium to stimulate responsive underlying tissues than PAMPs that remain attached to whole bacteria, we next aimed to determine which major classes of bacteria may contribute to the soluble PAMP pools in the gut. We first confirmed that each bacterial strain examined stimulated macrophage TNF-α production in response to direct contact with intact organisms (Figure 7A). However, while filter-sterilised culture supernatants of enterobacterial species such as *E. coli*, *S. typhimurium* and *P. aeruginosa* stimulated macrophage TNF-α production at dilutions from 1∶1,000 to 1∶10,000, those of four Gram-positive organisms (*L. plantarum*, *B. bifidum*, *E. faecalis* and *S. aureus*), or the Gram-negative *B. fragilis*, stimulated macrophages only at a dilution of 1∶10, or not at all (Figure 7B). Soluble stimulants of TLR4 and TLR5 were found to be shed by *E. coli*, *S. typhimurium* and *P. aeruginosa*, but not *B. fragilis* or the Gram-positive organisms examined (Figure 7C). Next, using TLR2-transfectants to examine bacterial shedding of lipopeptides, we found that while the enterobacterial species examined are prolific shedders of lipopeptide, Gram-positive organisms released little or no soluble TLR2-stimulants into their surroundings and *B. fragilis* displayed an intermediate phenotype (Figure 7D).

## Discussion

It is now widely accepted that the destructive cycles of inflammation observed in IBDs are potentiated by inappropriate responses towards the host intestinal microbiota [1]. However, it is not yet clear how the gut remains tolerant of the inflammatory molecules expressed by the commensal microbiota in health, nor why this tolerance should become broken during episodes of disease. Recent evidence suggests that the stimulation of Toll-like receptors (TLRs) by PAMPs expressed and released by intestinal bacteria may contribute to the propagation of IBDs [2], [7]–[12], [31], [32], leading to the suggestion that elevated intestinal PAMP concentrations could represent a risk factor for disease progression [10]–[12], [32]. As very little information is currently available regarding PAMP concentrations in the luminal contents of the healthy and diseased gut, or of which major bacterial groups may contribute to the intestinal PAMP pools, we sought to employ a novel bioassay-based approach to address these outstanding questions.

Our results reveal for the first time that the induction of colitis or ileitis in mice is associated with marked increases in the luminal concentrations of PAMPs specific for TLR2, TLR4 and/or TLR5. Moreover, we found that these changes were disease-specific; ileitis being associated with marked increases in concentrations of lipopeptide-, LPS- and flagellin-equivalents while colitis was associated with a modest increase in lipopeptide concentrations, a large increase in LPS and no change to flagellin concentrations. Such marked differences in intestinal PAMP concentrations are suggestive of major shifts in the relative abundances of the bacterial groups that make up the intestinal flora. Accordingly, many previous studies have reported that the microbiota can change markedly during IBDs in human subjects and in animal models [20]–[24]. In particular, with respect to the specific models examined here (*ie T. gondii*-induced ileitis and DSS-induced colitis), we have shown previously using both culture-dependent and culture-independent methods that a reduction in bacterial diversity and a dramatic increase in the numbers of enterobacterial species occurs in both conditions [11]–[13].

The relevance of the latter finding is highlighted by the present study, as we show that enterobacterial species are likely to represent not only dominant contributors to the pool of soluble TLR4 stimulants in the gut, but also major contributors to the pools of soluble pro-inflammatory and TLR2-stimulating agents in the gut. This conclusion is drawn from the observation that several members of the other numerically major Gram-negative group, *Bacteroides* species, did not stimulate TLR4-signalling (Figure 6C). Moreover, we found that the shedding of pro-inflammatory stimulants by enterobacterial species was several orders of magnitude higher than that observed from *Bacteroides* species and Gram-positive commensal organisms such as *Lactobacilli* and *Bifidobacteria* (Figure 7B, D). Taken together, these findings suggest that despite their numerically minor presence in the gut, the enterobacteria may nevertheless represent prominent contributors to the intestinal pools of potentially pro-inflammatory agents. The *in vitro* and *in vivo* findings of the present study therefore support previous observations of a large expansion of enterobacterial numbers in both DSS-induced colitis and *T. gondii*-induced ileitis [11]–[13]. In particular, our previous culture analysis of the microbiota of the inflammed colon suggested that numbers of Bacteroides/Prevotella spp., enterococci, clostridia and lactobacilli were not significantly altered by DSS-treatment, while enterobacterial species expanded by several orders of magnitude, further suggesting that the increase in TLR2 and TLR4 stimulants is driven by the overgrowth of enterobacterial species, rather than other groups [13]. In the *T. gondii*-induced ileitis model, 16S gene sequence analysis revealed that ileal lactobacilli, bifidobacteria and clostridia disappeared and were replaced with enterobacterial and bacteroides species, suggesting that enterobacterial overgrowth drives not only the increase in TLR2 and TLR4 stimulants in this model, but also the increase in TLR5-stimulants [12].

It should be noted that the PAMP-profiling assay we have described has specific advantages and limitations in comparison with alternative methods of examining alterations to the intestinal microbiota. For example, although the assay is culture-independent, requires no knowledge of target organism DNA-sequences and is not biased towards particular groups arising from primer design, cloning efficiency or antibody-specificity, it cannot provide information regarding specific groups or species of bacteria or the exact molecular species contributing to each TLR-signal. For this reason, and as there is some debate as to the diversity of ligands that may be recognised by TLR2 and TLR4 [28], we aimed to clarify which types of molecules may be responsible for the stimulation of these TLRs in SFE. Since both lipid-IVa and polymyxin-B completely blocked TLR4 signalling induced by SFE, it seems likely that LPS is the only TLR4 stimulant present in SFE (Figure 5B). However, as we found that lipases blunted, but did not eliminate, the TLR2-signal induced by SFE (Figure 5A), we cannot exclude the possibility that other proposed TLR2 stimulants, such as lipoteichoic acid or *Bacteroides* LPS may also contribute to the TLR2-signal induced by SFE [33], [34]. Thus, although we note that others have proposed that contaminating lipopeptides may account for the previously reported TLR2-stimulating properties of peptidoglycan and lipoteichoic acid [35], [36], we refer to the TLR-stimulants measured in each assay as “PAMP-equivalents”, rather than *de facto* lipopeptides or lipopolysaccharides.

A key question that remains to be addressed in future studies is whether increased concentrations of luminal PAMPs serve merely as markers, or as mediators of IBDs. Evidence that they may serve as disease mediators is present in our earlier observations that oral administration of *E. coli* lipid-A markedly promotes *T. gondii*-induced ileitis in mice, while neutralisation of intestinal LPS by treatment with polymyxin-B reduced the severity of ileitis [11], [12]. Likewise, rectal administration of LPS was shown to promote colitis in rabbits [8], and administration of the TLR9 agonist CpG-ODN enhanced DSS-induced colitis in mice [10]. Furthermore, rectally applied *S. typhimurium* flagellin was found to aggravate colonic inflammation and increase mortality in DSS-induced colitis [37]. However, the data obtained from studies of mice deficient in TLRs suggest a more complex role for PAMP recognition in the gut, as both barrier protective [4]–[6], [17]–[19] and pro-inflammatory roles [7], [8], [11]–[16], [32] for TLR-mediated PAMP recognition in the gut have been reported.

One possible way of conciliating these views is to suggest that while PAMP sensing by epithelial cells serves to enhance barrier function, if the barrier eventually fails and PAMPs reach the more sensitive underlying cells and tissues, inflammation may be triggered to protect the host from translocating bacteria. This notion is supported by the fact that while the intact colon is largely unresponsive to exogenously applied flagellin, LPS or lipoteichoic acid [9], [37]–[39], when the epithelial barrier is disrupted by agents such as DSS, diverse PAMPs can trigger overt inflammation and damage [10]–[12], [37], [38]. Furthermore, as we found that high concentrations of PAMPs are generated in the ileum of *T. gondii*-infected mice, yet colonic inflammation is not observed despite the fact that these PAMPs should pass to the colons of these mice, our data further support the notion that even very high concentrations of PAMPs are not alone sufficient to induce intestinal inflammation when the epithelial barrier is intact. Studies involving cell-type specific deletion of TLRs, rather than systemic knockouts as currently reported, will likely be required to shed further light on this complex issue.

In summary, we conclude that while the healthy gut contains appreciable quantities of soluble ligands of TLR2, TLR4 and TLR5, the relative abundances of stimulants of these receptors can increase dramatically during the course of murine IBDs. Future studies are warranted to establish whether such alterations to PAMP profiles are also observed in human IBDs, such as ulcerative colitis and Crohn's disease, and whether such alterations serve as markers or as mediators of these diseases. If the latter turns out to be evident, studies are also warranted to investigate whether normalisation of intestinal PAMP concentrations may possess therapeutic potential for these diseases.
